# Establishment of a novel classification system for alveolar morphology in infants with unilateral complete cleft lip and palate

**DOI:** 10.1007/s00784-023-05353-z

**Published:** 2023-10-27

**Authors:** Haizhen Li, Yuxia Hou, Qingnan Mou, Zhanping Ren, Yongwei Tao, Yuhua Jiao, Huimei Huang, Huaxiang Zhao

**Affiliations:** 1 Key Laboratory of Shaanxi Province for Craniofacial Precision Medicine Research, College of Stomatology, Xi’an Jiaotong University, No. 98, Xiwu Road, Xincheng District, Xi’an, Shaanxi People’s Republic of China; 2 Department of Orthodontics, Shanghai Ninth People’s Hospital, College of Stomatology, Shanghai Jiao Tong University School of Medicine, Shanghai, People’s Republic of China; 3 Department of Orthodontics, College of Stomatology, Xi’an Jiaotong University, Xi’an, Shaanxi People’s Republic of China; 4 Department of Cleft Lip and Palate Surgery, College of Stomatology, Xi’an Jiaotong University, Xi’an, Shaanxi People’s Republic of China; 5 Department of Nephrology, Xi’an Children’s Hospital, The Affiliated Children’s Hospital of Xi’an Jiaotong University, Xi’an, Shaanxi People’s Republic of China

**Keywords:** Unilateral complete cleft lip and palate, Presurgical nasoalveolar molding, Alveolar morphology, Classification system, Mathematical clustering

## Abstract

**Objectives:**

Unilateral complete cleft lip and palate (UCCLP) is one of the most severe clinical subtypes among cleft lip and palate (CLP), making repair surgery and subsequent orthodontic treatment particularly challenging. Presurgical nasoalveolar molding (PNAM) has shown conflicting and heterogeneous results in the treatment of UCCLP patients, raising questions about whether the diversity in alveolar anatomical morphology among these patients plays a role in the effectiveness of PNAM treatment.

**Materials and methods:**

We collected 90 digital maxillary models of infants with UCCLP and performed mathematical clustering analysis, including principal component analysis (PCA), decision tree modeling, and area under the ROC Curve (AUC) analysis, to classify alveolar morphology and identify key measurements. We also conducted clinical evaluations to assess the association between the alveolar morphology and CLP treatment outcomes.

**Results:**

Using mathematical clustering analysis, we classified the alveolar morphology into three distinct types: average form, horizontal form, and longitudinal form. The decision tree model, AUC analysis, and comparison analysis revealed that four measurements (Trans *AC*_*G*_-*AC*_*L*_, *M*_*L*_ length, *M*_*G*_ length and *Inc* length) were essential for clustering the alveolar morphology of infants with UCCLP. Furthermore, the blinded clinical evaluation indicated that UCCLP patients with alveolar segments of horizontal form had the lowest treatment outcomes.

**Conclusion:**

Overall, our findings establish a novel quantitative classification system for the morphology of alveolar bone in infants with UCCLP and suggest that this classification may be associated with the outcomes of CLP treatment.

**Clinical relevance:**

The multidisciplinary CLP team should thoroughly evaluate and classify the specific alveolar morphology when administering PNAM to infants with UCCLP.

**Supplementary Information:**

The online version contains supplementary material available at 10.1007/s00784-023-05353-z.

## Introduction

Cleft lip and/or palate (CL/P) is the most commonly occurring congenital craniofacial malformation worldwide, with a prevalence ranging from 1/500 to 1/2500 in newborn infants [1, 2]. Patients with this disorder not only face physical appearance disharmony but also experience difficulties with oral functions, such as speaking and swallowing, which can significantly impact their quality of life [3]. Of all the subtypes of CL/P, unilateral complete cleft lip and palate (UCCLP), accompanied by a complete discontinuity of the alveolar bone and upper lip extending to the nasal floor, poses the greatest risk for facial asymmetry and severe dental arch abnormalities. The complex nature of UCCLP makes surgical repair and subsequent orthodontic treatment particularly challenging [4, 5].

Presurgical nasoalveolar molding (PNAM) is an orthodontic treatment that typically starts within the first month after birth and before surgical repair for severe cleft lip and palate (CLP), particularly in infants with UCCLP. It capitalizes on the pliability of an infant’s cartilage and allows passive remodeling of the growing bony alveolus to reshape the severely displaced alveolar segments and nose. This treatment helps to achieve optimal alignment of the cleft alveolar segments and lips before surgical repair, reducing tension during the procedure and making surgery less difficult [6].

However, there is controversy regarding the effectiveness of the PNAM in the treatment of UCCLP [7]. While some studies have shown positive therapeutic effects of PNAM treatment on the dental arch form and the symmetry of the nose and lips of infants with UCCLP [8–11], other studies have reported no significant benefits from this treatment [12]. In fact, some studies have even reported negative effects of PNAM treatment [13].

A previous study has observed that the anatomy and alveolar morphology of unilateral cleft lip and palate vary among patients, and therefore, the PNAM treatment should be customized accordingly [14]. This finding is consistent with our clinical experience, leading us to propose that the conflicting and heterogeneous results regarding the efficacy of PNAM treatment for UCCLP may be due to the diverse clinical anatomy and alveolar morphology among patients. However, there is currently a lack of a standardized quantitative classification system for the morphology of alveolar bone in infants with UCCLP [14, 15], which makes it difficult to precisely utilize PNAM treatment and may contribute to the controversy regarding its effectiveness.

In the present study, we recruited a sample of 90 infants with UCCLP and obtained their digital dental models for analysis. Through measurements and mathematical cluster analysis, we identified three classifications of alveolar morphology in infants with UCCLP and determined the key measurement for classification. Furthermore, a blinded clinical evaluation with a small sample size was conducted, which revealed that the horizontal form of the alveolar segments has the lowest evaluation in PNAM treatment.

## Materials and methods

### Ethics statement

This study was approved by the Ethics Committee in Hospital of Stomatology, Xi’an Jiaotong University (No. xjkqll[2019]NO.003), and informed consent was obtained from the guardians of participants.

### Participants

We retrieved records of UCCLP patients who visited the Hospital of Stomatology, Xi’an Jiaotong University, from 2010 September to 2018 June. The inclusion criteria were as follows: (1) infants no older than 1 month after birth; (2) diagnosed with non-syndromic unilateral complete cleft lip and palate, either left or right side; (3) maxillary plaster or digital dental model recorded prior to any medical interventions. The exclusion criteria were as follows: (1) patients with other orofacial abnormalities or facial trauma; (2) patients who had received any medical interventions such as PNAM or plasticity with elastic bandage.

Overall, a total of 90 infants were selected to participate in the study, comprising 42 boys and 48 girls, with an average age of 16 ± 5 days.

### Measurements of alveolar morphology

To initiate the measurement process, we scanned all maxillary dental casts and converted them into digital models. These digital models were subsequently imported into the Geomagic software (3D Systems Inc., USA) for further analysis and measurements.

**Fig. 1:**
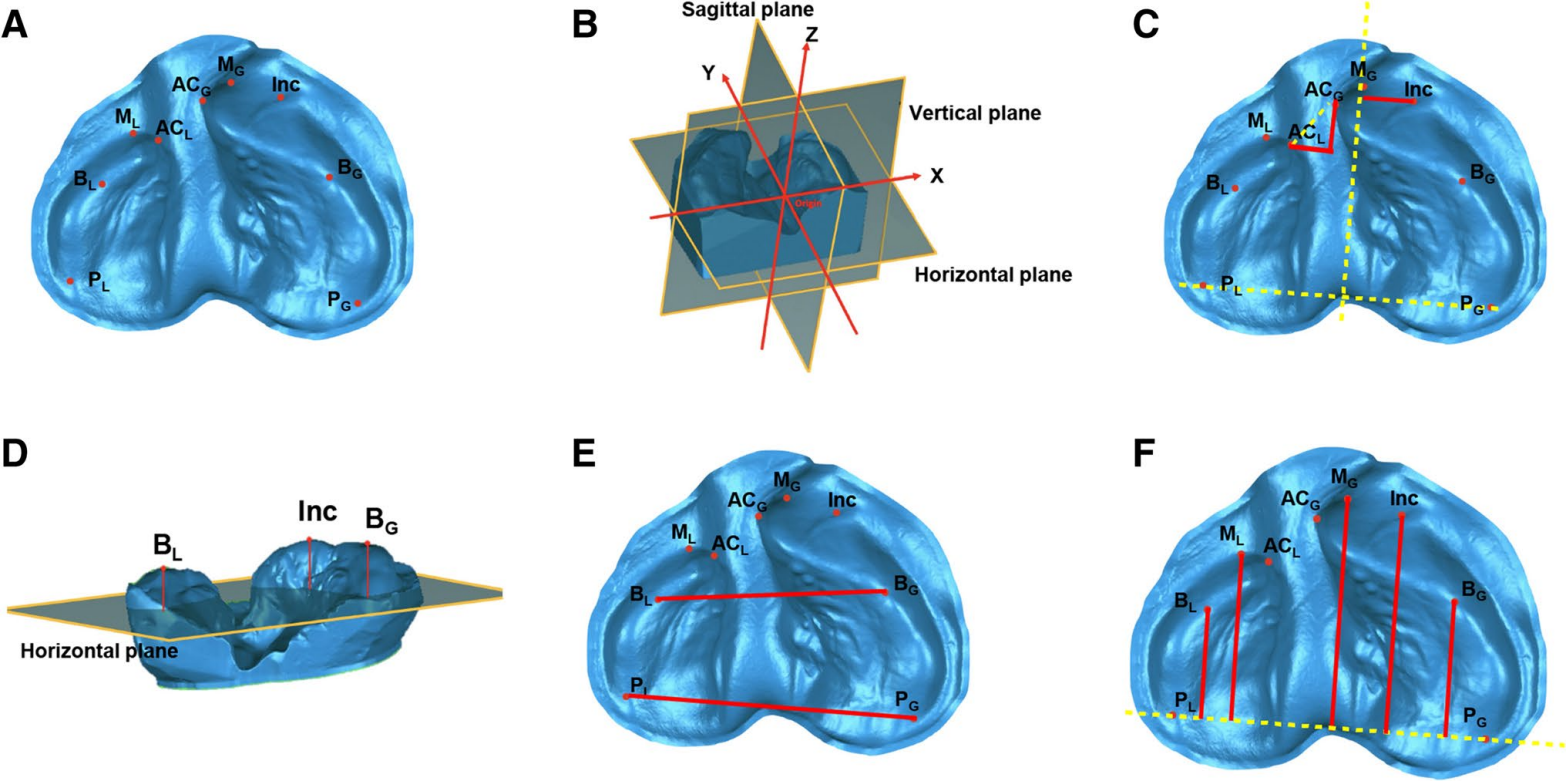
Illustration of measurements of alveolar morphology in infants with UCCLP. **A** Illustration of landmarks used in the study (detailed definition of the landmarks are listed in Supplemental Table 1). **B** Illustration of the three-dimensional system of coordinates used in the study. Origin (the original point): the midpoint between point *P*_*G*_ and *P*_*L*_. The horizontal plane: constructed using points *P*_*G*_, *P*_*L*_, and *M*_*G*_. The sagittal plane: established as a perpendicular plane passing through the midpoint of the line between points *P*_*G*_ and *P*_*L*_. The coronal plane: created by a plane that was perpendicular to the horizontal and sagittal planes and passed through the point Origin. **C**–**F** Transverse, sagittal, and vertical items of measurements used in the study (detailed definitions of measurements are listed in Supplemental Table 2)

Based on previous studies [16], specific landmarks were identified on the alveolar bones using anatomic structures (Fig. 1A; Supplemental Table 1). Subsequently, a three-dimensional system of coordinates was constructed (Fig. 1B). Briefly, the original point (Origin) was defined as the midpoint between point *P*_*G*_ and *P*_*L*_. The horizontal plane was constructed using point *P*_*G*_, *P*_*L*_, and *M*_*G*_. The sagittal plane was established as a perpendicular plane passing through the midpoint of the line between points *P*_*G*_ and *P*_*L*_. Finally, the coronal plane was created by a plane that was perpendicular to the other two reference planes and passed through the point Origin.

Afterward, various length measurements, including transverse, sagittal, and vertical items, were taken (Fig. 1C–F; Supplemental Table 2).

To assess the replicability and reliability of the measurements, we randomly selected 18 participants and performed the intra-class correlation coefficient (ICC) test. The measurements were taken repeatedly after a 2-week interval, and the ICC value ranged from 0.95 to 1, indicating high reliability and reproducibility of the measurements.

### Clustering methods for alveolar morphology

To cluster and classify various alveolar morphologies and determine the key measurements for classification, we firstly performed principal component analysis (PCA) using the PRCOMP function in the R Package to reduce the dimensionality of the measurement items. Next, we generated a heatmap using the PHEATMAP function from the R package to visualize the scaled data. Additionally, we constructed a decision tree model with the RPART function from the R package. To identify the critical factors influencing the classification of alveolar morphology, we conducted an analysis of area under the ROC Curve (AUC) with a one-by-one adding and one-by-one removal strategy.

After categorizing the alveolar morphology in patients with UCCLP, the value of key items among various classifications was compared using *t* tests, and the *P* values were subjected to Bonferroni correction.

### Evaluation of the effectiveness of PNAM on various classifications of alveolar morphology

To investigate whether the alveolar morphology affects the therapeutic efficacy of PNAM, a clinical evaluation was conducted. Out of the initial 90 UCCLP patients, 15 underwent a complete PNAM treatment followed by cleft lip and palate repair, all handled by the same orthodontic and orthopedic team. In detail, infants diagnosed with UCCLP began their PNAM treatment by Dr. Yuxia Hou within 4 weeks after birth. Subsequently, the repair surgery for UCCLP, employing the modified Mohler rotation-advancement cheiloplasty and two-flap palatoplasty techniques, was performed by Dr. Zhanping Ren (the senior surgeon) and Dr. Yongwei Tao (the junior surgeon). When these patients reached the age of 3 ~ 4 years, two experienced specialists in orthodontics and orthopedics conducted an evaluation of the treatment outcomes. This evaluation was based on the self-made criteria which included assessments of facial esthetics and dental arch morphology (Supplemental Table 3). The evaluators remained anonymous and blinded to the patients’ initial alveolar morphology and treatment history. We have summarized our entire research rationale in a flow chart (Supplemental Fig. 1).

## Results

### Mathematical cluster analysis classifies the alveolar morphology of infants with UCCLP into three distinct types

**Fig. 2:**
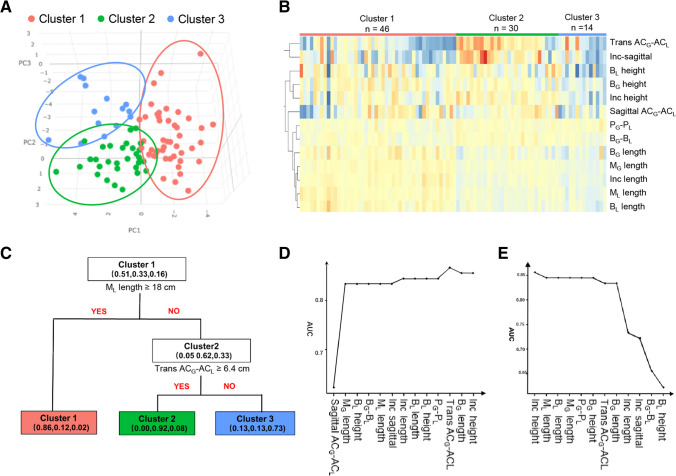
The alveolar morphology of infants with UCCLP can be classified into three distinct clusters using mathematical cluster analysis. **A** The three-dimensional scatter plot of principal component analysis (PCA) can categorize the alveolar morphology of 90 infants with UCCLP into three clusters. Each dot represents the alveolar morphology of one infant. **B** The heatmap of the measurements of alveolar morphology for the three clusters. The colors on the heatmap represent the relative values of the measurements, with warmer colors indicating higher values and cooler colors indicating lower values. **C** The decision tree model shows *M*_*L*_ length and Trans *AC*_*G*_-*AC*_*L*_ are the most important parameters for differentiating the three clusters with high accuracy. **D, E** Alterations in AUC when adding **(D)** and removing **(E)** the measurements one by one

During clinical practice, we have observed that infants with UCCLP exhibit distinct shapes in their maxillary alveolar casts. To classify the alveolar morphology in a scientific manner, we obtained measurements of the maxillary dental arch from 90 infants with UCCLP. We then utilized the PCA, a commonly used method for reducing dimensionality and clustering objects into different groups [17, 18], to identify patterns and relationships between variables in the dataset and classify the alveolar morphology into different types. The result of PCA showed that the dataset of alveolar morphology could be categorized into three distinguishable clusters (Fig. 2A).

Among the identified three clusters of alveolar morphology, cluster 1 had the highest proportion, accounting for 51.11% of the total samples, while cluster 2 and cluster 3 accounted for 33.33% and 15.56% of the total samples, respectively (Fig. 2A and B). The comparison analysis revealed that cluster 2 had greater transverse measurements, such as trans *AC*_*G*_-*AC*_*L*_ and *Inc*-sagittal, than both cluster 1 and cluster 3 (Table 1), indicating that alveolar segments in cluster 2 were wider clinically. On the other hand, cluster 3 displayed the greatest measurements in major items of sagittal direction such as *M*_*G*_ length, *M*_*L*_ length, *B*_*G*_ length, *B*_*L*_ length, and *Inc* length (Table 1), suggesting that cluster 3 had the longest segments of alveolar bones.

**Table 1:** Measurements of alveolar morphology in three clusters

Items (mm)		Cluster 1	Cluster 2	Cluster 3	*P* (Cluster 1 vs 2)	*P* (Cluster 1 vs 3)	*P* (Cluster 2 vs 3)
Transverse	P_G_-P_L_	34.58 ± 3.3	34.55 ± 2.62	33.13 ± 3.71	1.000	0.477	0.229
	B_G_-B_L_	28.65 ± 3.28	30.7 ± 3.1	29.24 ± 3.55	0.195	1.000	0.209
	Trans AC_G_-AC_L_	5.34 ± 3.53	8.29 ± 3.28	4.74 ± 3.25	0.022^*^	1.000	0.000^***^
	Inc-Sagittal	3.12 ± 1.72	5.69 ± 2.22	3.32 ± 2.16	0.001^**^	1.000	0.000^***^
Sagittal	Sagittal AC_G_-AC_L_	4.2 ± 1.97	5.89 ± 1.54	5.42 ± 2.56	0.060	0.214	1.000
	M_G_ length	23.45 ± 2.73	26.72 ± 2.14	29.19 ± 2.79	0.001^**^	0.000^***^	0.000^***^
	M_L_ length	16.2 ± 3.43	17.08 ± 1.74	20 ± 2.37	0.768	0.000^***^	0.000^***^
	B_G_ length	11.8 ± 3.8	12.95 ± 2.62	15.34 ± 2.63	0.653	0.000^***^	0.002^**^
	B_L_ length	12.44 ± 2.78	13.11 ± 1.75	15.53 ± 2.65	1.000	0.000^***^	0.000^***^
	Inc length	17.57 ± 2.69	19.74 ± 2.08	22.69 ± 2.72	0.030^*^	0.000^***^	0.000^***^
Vertical	B_G_ height	3.79 ± 0.99	4.97 ± 1.06	5.19 ± 1.32	0.010^*^	0.001^**^	1.000
	B_L_ height	2.34 ± 1.79	3.8 ± 1.18	3.72 ± 1.6	0.012^*^	0.011^*^	1.000
	Inc height	3.45 ± 1.16	5.35 ± 0.84	4.6 ± 1.72	0.000^***^	0.028^*^	0.080^*^

*, *P* < 0.05; **, *P* < 0.01; ***, *P* < 0.001 (The *P* value had been performed with Bonferroni correction)

**Fig. 3:**
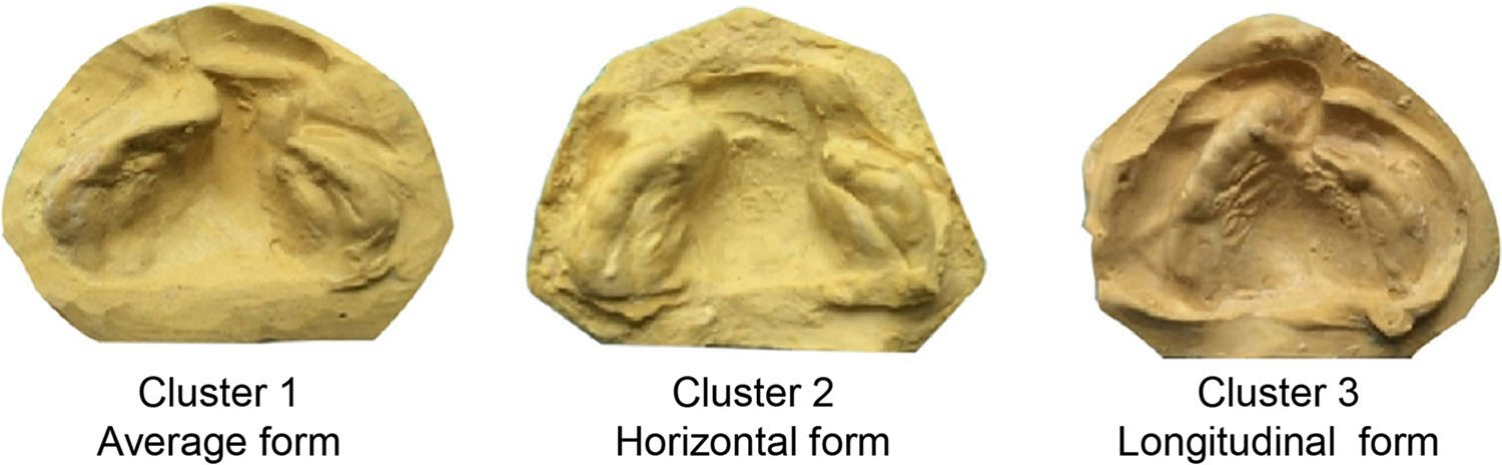
The represented forms of the three classifications of alveolar morphology for infants with UCCLP. Cluster 1 is represented by the average form, cluster 2 is represented by the horizontal form, and cluster 3 is represented by the longitudinal form

After performing the mathematical analysis, it was observed that cluster 2 exhibited the widest alveolar segments, while cluster 3 exhibited the longest. Therefore, we identify three classifications of alveolar morphology of infants with UCCLP: cluster 1 as average form, cluster 2 as horizontal form, and cluster 3 as longitudinal form (Fig. 3).

### Critical measurements for classifying the alveolar morphology of infants with UCCLP

In the next work, we aimed to identify key measurements for classifying alveolar morphology in infants with UCCLP. The decision tree model showed *M*_*L*_ length and Trans *AC*_*G*_-*AC*_*L*_ were the most important parameters for differentiating the three clusters with high accuracy (Fig. 2C). To assess the diagnostic efficacy of the measurements, we used AUC analysis. The one-by-one adding process showed that *M*_*G*_ length was the most critical measurement for diagnosis, with an AUC value of over 0.8 when *M*_*G*_ length was added (Fig. 2D). Similarly, removing *Inc* length and *B*_*G*_-*B*_*L*_ in the one-by-one removal strategy resulted in a significant decline in the AUC value (Fig. 2E).

In the comparison analysis of the measurements, we observed that there was no significant difference in *B*_*G*_-*B*_*L*_ item among the three clusters. Trans *AC*_*G*_-*AC*_*L*_ was effective in distinguishing between cluster 2 and cluster 1/cluster 3, while *M*_*L*_ length was useful in differentiating cluster 3 from cluster 1/cluster 2. Additionally, *M*_*G*_ length and *Inc* length showed statistically significant differences among all three clusters (Table 1).

Based on the findings, it appears that the most crucial measurements for classifying the alveolar morphology in infants with UCCLP are Trans *AC*_*G*_-*AC*_*L*_, *M*_*L*_ length, *M*_*G*_ length, and *Inc* length.

### Most alveolar segments of horizontal form receive poor scores when evaluating treatment effects

To further explore the potential sensitivity of different classifications of alveolar morphology to PNAM treatment, a clinical evaluation was conducted. The results revealed that 80% of alveolar segments classified as horizontal form scored poorly. In contrast, 33.3% of average form or longitudinal form alveolar segments achieved a good rating, and 66.7% reached a general degree (Table 2).

**Table 2:** The scoring results of specialist in evaluating the treatment effect

Cluster	Cluster 1 Average form	Cluster 2 Horizontal form	Cluster 3 Vertical form
Level			
Good	33.3%	20%	33.3%
General	66.7%	0%	66.7%
Bad	0%	80%	0%

## Discussion

UCCLP is a challenging clinical subtype of cleft lip and/or palate that poses difficulties for repair surgery and subsequent orthodontic treatment [19, 20]. PNAM has been implemented to alleviate the challenges of repair surgery, with the aim of achieving better outcomes in terms of facial symmetry and coordination of dental arch [6, 20, 21]. However, the heterogeneity of outcomes of PNAM treatment has made it difficult to reach a consensus on its efficacy [7, 8, 12, 13, 21]. Drawing on previous studies [14, 15] and our clinical experience, we hypothesize that there may be distinct alveolar morphologies among infants with UCCLP, which may contribute to the conflicting results regarding the effectiveness of PNAM.

In this study, we made three main findings. First, the mathematical analysis classifies the alveolar morphology of infants with UCCLP into three distinct types: average form, horizontal form, and longitudinal form. Second, we found that specific measurements, including Trans *AC*_*G*_-*AC*_*L*_, *M*_*L*_ length, *M*_*G*_ length, and *Inc* length, are essential for clustering the alveolar morphology of infants with UCCLP. Finally, the preliminary blinded clinical evaluation indicates that alveolar segments classified as horizontal form may have the lowest effectiveness when treated with PNAM.

After obtaining at least ten measurements of alveolar morphology in 90 infants, we encountered difficulties to classify the dental casts into different types due to the complexity of the data. To overcome this challenge, we adopted the method of PCA clustering, which is widely used in biomedical studies, including ours [22], but is not commonly utilized in clinical dental studies. The advantage of PCA clustering is that it can effectively handle high-dimensional data by reducing its dimensions, making it easier to interpret and visualize [23]. Additionally, it can minimize any subjective bias during clustering [24]. Using PCA clustering, we identified three classifications of alveolar segments in patients with UCCLP, each exhibiting distinct clinical morphology (Figs. 2A and 3). Significant differences were also observed among measurements of the three identified types (Table 1). However, it is important to note that PCA clustering is not always a universal solution and depends on the variables measured for each sample. Therefore, measurements relying on clinical experience are still critical for further analysis and classification in the future.

We found that UCCLP patients classified into horizontal form alveolar segments exhibited the worst long-term outcomes in terms of facial esthetics and dental arch morphology, despite receiving the same PNAM treatment as those classified into the other two classifications (Table 2). In addition, we noted that the items representing the width of the fissure of separated segments (Trans *AC*_*G*_-*AC*_*L*_ and *Inc*-sagittal) in horizontal form (cluster 2) were greater than those in average form and longitudinal form (Table 1). We hypothesize that it requires greater movement to realign deviated alveolar segments in the horizontal form compared to the other two types of alveolar morphology, impacting the effectiveness of PNAM treatment. However, it remains unclear whether other factors, such as genetic variations in cartilage plasticity, affect treatment outcomes, and further investigation is needed.

Several limitations of this study should be mentioned. First, the clinical evaluations were based on self-developed criteria rather than established objective criteria such as the Goslon Yardstick [25]. This may hinder comparisons in multi-center studies. Second, there are potential confounders to consider. While the same orthodontist and orthopedic team performed the PNAM treatment and CLP repair surgery, variability remains in aspects of the treatment, such as initial UCCLP conditions and different treatment experience at different time. Lastly, the limited sample size may restrict the generalizability of the results. To address these limitations, a clinical evaluation with a larger sample size or a prospective study should be considered across multiple centers, using objective criteria, to analyze the association between alveolar morphology and the effectiveness of PNAM treatment.

### Supplementary Information

Below is the link to the electronic supplementary material.

Supplementary file1 (PDF 421 KB)

## Data Availability

More additional information are available from the corresponding author Dr. Huaxiang Zhao (huaxiangzhao@xjtu.edu.cn) on reasonable request.
