# Supplementary material for: Establishment of a novel classification system for alveolar morphology in infants with unilateral complete cleft lip and palate
Source: Clin Oral Investig. 2023 Oct 27;27(12):7643–50. doi: 10.1007/s00784-023-05353-z (PMC10713668; doi:10.1007/s00784-023-05353-z)
Supplement: Supplementary file 1 — Supplementary file1 (PDF 421 KB) [file 784_2023_5353_MOESM1_ESM.pdf]

Supplemental materials for

Establishment of a novel classification system for alveolar morphology in infants with unilateral complete cleft lip and palate

The Supplemental materials includes:

- ✧ **Supplemental Figure 1.** The flow chart summarizing our research rationale
- ✧ **Supplemental Table 1.** Definition of the landmarks used in this work
- ✧ **Supplemental Table 2.** Definitions of measurements used in this study
- ✧ **Supplemental Table 3.** The self-made criteria used for assessing treatment outcomes

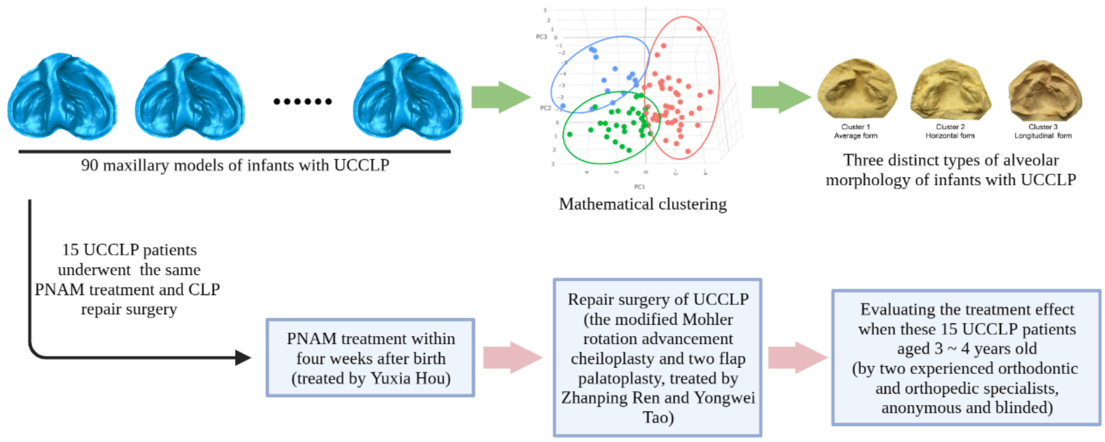

Supplemental Figure 1. The flow chart summarizing our research rationale.

Supplemental Table 1. Definition of the landmarks used in this work

Landmarks	Definition
P_G	The posterior end-point of the alveolar crest in the greater segment
P_L	The posterior end-point of the alveolar crest in the lesser segment
AC_G	The anterior end-point of the alveolar crest in the greater segment
AC_L	The anterior end-point of the alveolar crest in the lesser segment
B_G	The intersection point between the palatally extended buccal frenum sulcus line and the palatal gingival groove in the greater segment
B_L	The intersection point between the palatally extended buccal frenum sulcus line and the palatal gingival groove in the lesser segment
M_G	The most anterior point of the greater segment from P_G-P_L line
M_L	The most anterior point of the lesser segment from P_G-P_L line
Mid	The midpoint of the P_G-P_L line
Inc	The intersection point between the labial frenum-incisive papilla point line and the alveolar crest of the premaxilla in the greater segment

Supplemental Table 2. Definitions of measurements used in this study

Measurements	Definition
P_G-P_L	Distance between P_G in the GS and P_L points in the LS
B_G-B_L	Distance between B_G in the GS and B_L points in the LS
Trans AC_G-AC_L	Transverse cleft gap between AC_G in the GS and AC_L in the LS
Sagittal AC_G-AC_L	Sagittal cleft gap between AC_G in the GS and AC_L in the LS
M_G Length	Length of M_G segment in the GS and P_G-P_L
M_L Length	Length of M_L segment in the LS and P_G-P_L
B_G Length	Length of B_G segment in the GS and P_G-P_L
B_L Length	Length of B_L segment in the GS and P_G-P_L
Inc Length	Length of Inc segment in the GS and P_G-P_L
Inc-Sagittal	Perpendicular distance from Inc point to sagittal line
M_G height	Distance from point M_G to the horizontal reference plane
M_L height	Distance from point M_L to the horizontal reference plane.
Inc height	Distance from point Inc to the horizontal reference plane.

GS, Greater segment; LS, lesser segment

Supplemental Table 3. The self-made criteria used for assessing treatment outcomes

Items	Rating	Descriptions
Facial aesthetics	Good	No need for a second reconstructive surgery or a simple second reconstructive surgery to achieve good nasolabial aesthetics.
	General	After a complex second reconstructive surgery, nasolabial aesthetic improvement may be achieved.
	Bad	Even after a complex second reconstructive surgery, achieving good nasolabial aesthetics is challenging.
Dental arch morphology	Good	The dental arch is basically symmetrical.
	General	After simple orthodontic treatment, a good dental arch morphology can be obtained.
	Bad	Even after complex orthodontic treatment, the dental arch morphology remains poor.

Overall evaluation criteria:

- Good: At least one item is rated "Good" and the other is either "Good" or "General".
- General: Both items are rated "General" or they have mixed ratings of "Good" and "Bad".
- Bad: At least one item is rated "Bad" and the other is either "Bad" or "General".
